# Teratoid Cyst of the Postauricular Region: The First Ever Case Report

**DOI:** 10.1155/2017/9235925

**Published:** 2017-12-12

**Authors:** Sabin Ranabhat, Mamata Tiwari, Sushna Maharjan

**Affiliations:** Department of Pathology, Chitwan Medical College, Bharatpur, Nepal

## Abstract

Rudolf Virchow is considered to be the first scientist to have used the word sebaceous cyst. It was thought that these lesions occurred due to retention of sebaceous secretion consequent to obstruction of sebaceous ducts of sebaceous glands, although that was found not to be the case. In all these cysts, the cavity is filled with keratin. There are six types of keratin-filled cysts, namely, epidermoid, dermoid, teratoid, keratinous, trichilemmal, and teratoma cyst, which have one common name “dermoid cyst.” Of the six, teratoid cyst is the least common. In contrast to other dermoid cysts, teratoid cysts contain tissue elements derived from all the three germ layers, namely, ectoderm, mesoderm, and endoderm. Teratomas can be differentiated from teratoid cysts by the fact that recognizable organ structures may be found in the former; examples include teeth and skin. Teratoid cysts can develop anywhere in the body but rarely arise in the head and neck region. They have never been reported in the postauricular region. In this case report, we present a case of teratoid cyst in the postauricular region in a 21-year-old female. The significance of this case lies in its rarity.

## 1. Introduction

Keratin-filled cysts are often improperly referred to as sebaceous cysts because keratin debris of these cysts resembles sebaceous material on casual examination. Rudolf Virchow is considered to be the first scientist to have used the word sebaceous cyst. It was thought that these lesions occurred due to retention of sebaceous secretion consequent to obstruction of sebaceous ducts of sebaceous glands, although that was found not to be the case [1].

The following theories have been put forward regarding the origin of dermoid cysts: (i) There is congenital inclusion of dermal and epidermal elements of germ layers in deeper tissues along the embryonic lines of fusion. (ii) Implantation of dermal and epidermal elements of surface epithelium can occur after birth due to trauma, which may proliferate and keratinize. (iii) Growth can occur from the rest of totipotent stem cells displaced from the blastomere [2].

The term “epidermal cysts” was coined by Warvi and Gates in 1943 [3]. Meyer, in 1955, used the term “teratoid cyst” for the first time in his classification of keratin-filled cysts of floor of mouth into epidermoid, dermoid, and teratoid types. Of the three, teratoid cyst is the least common [4]. Since then, this classification has been applied to other parts of the body as well. Apart from these three, three more entities which are filled with keratinous debris can occur in the body, namely, teratoma, keratinous cyst, and trichilemmal cyst.

Epidermoid cysts, also known by various other names such as epidermal cyst, epidermal inclusion cyst, and follicular infundibular cyst, are the most common cutaneous cysts. They occur anywhere in the body, but the most common locations are face, scalp, neck, and trunk [5].

Dermoid cysts in the skin and subcutaneous tissue occur most commonly on the face, neck, or scalp [1]. These cysts are located in the midline in the head and neck region and sometimes on the floor of the mouth because they develop from entrapment of epithelial cells along the lines of embryonic closure [6].

Teratoid cysts can develop anywhere in the body but rarely arise in the head and neck region [7]. Keratinous cysts develop on the face, neck, upper trunk, labia majora, and scrotum [1, 2]. Trichilemmal or pilar cysts occur in 5–10% of the population. More than 90% occur on the scalp, where they are the most common cutaneous cysts [8].

Keratinous cysts are lined by laminated keratin and the cavity is filled with keratin and lipid debris. Lining epithelium is not present. These cysts may have disruption of the wall leading to acute inflammation and intense foreign body giant cell reaction in the underlying stroma. Epidermoid, dermoid, teratoma, and teratoid cysts are lined by keratinized stratified squamous epithelium with distinct granular layer. Trichilemmal cysts do not have granular layer. Dermoid cysts have skin adnexal structures in the wall in addition. Teratoid cysts may be lined by respiratory epithelium instead of squamous epithelium and contain tissue elements derived from all the three germ layers, namely, ectoderm, mesoderm, and endoderm in the wall. Teratomas can be differentiated from teratoid cysts by the fact that recognizable organ structures may be found in the former; examples include teeth, skin, and endocrine tissue. Tissues in teratomas are derived from one or more of three germ layers [9].

The importance of recognition of teratoid cyst is that complete excision should be done to prevent recurrence. Incomplete excision has been found to lead to recurrence even after 17 years [10]. Furthermore, malignant transformation can occur in teratoid cysts and teratoma.

## 2. Case Report

A 21-year-old Nepalese female presented with painless postauricular mass to the Ear, Nose and Throat (ENT) Department. It was first noticed when the patient was 6 years old. The lesion gradually increased in size over the years from a pea-sized nodule. At the age of 10 years, the nodule was drained of its contents by making a small nick into it at a local clinic by a paramedic. Obviously, the child's parents noticed the lesion to grow back gradually after one year. The lesion measured 2 × 1 × 1 cm when the patient presented to ENT OPD at the age of 21 years.

On examination, the mass was nontender and cystic on palpation. Punctum, sinus, scar, or any kind of discharge was not present on the surface. Pinna was pushed anteriorly by the mass and retroauricular sulcus was obliterated. Apart from the mass, the patient had no other positive findings. Dermoid cyst, Warthin's cyst, and parotid tail cyst were kept in the differential diagnoses.

Fine needle aspiration cytology was performed by a pathologist in the department of pathology. Pasty material was obtained from the lesion. Smears prepared from the material showed plenty of keratin debris and anucleate squames. Hair shafts were conspicuous by their absence. On the basis of these microscopic findings, cytological diagnosis was made as “dermoid cyst.”

**Figure 1:**
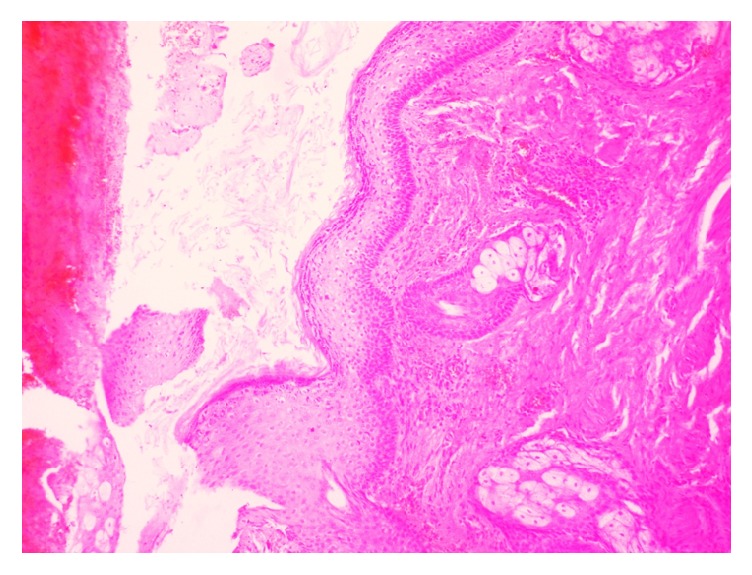
Cystic cavity lined by keratinized stratified squamous epithelium and the cavity filled with anucleate squames and keratin.

**Figure 2:**
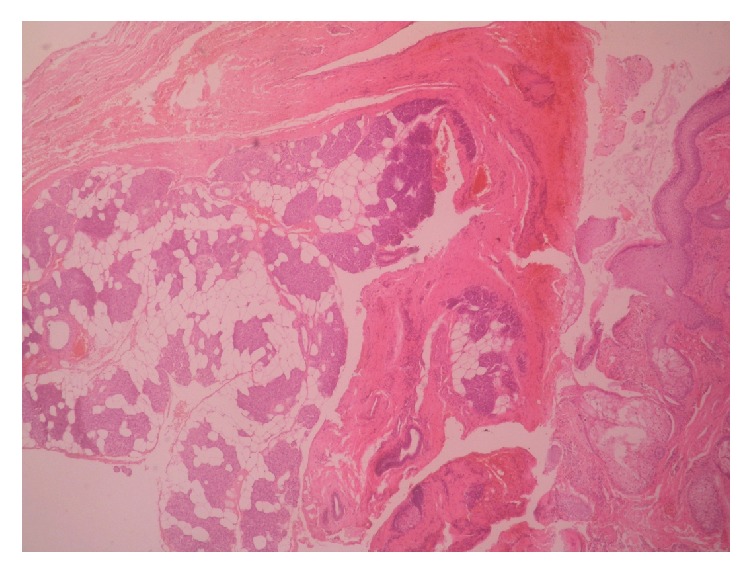
Cystic cavity lined by keratinized stratified squamous epithelium and the cavity filled with anucleate squames and keratin.

**Figure 3:**
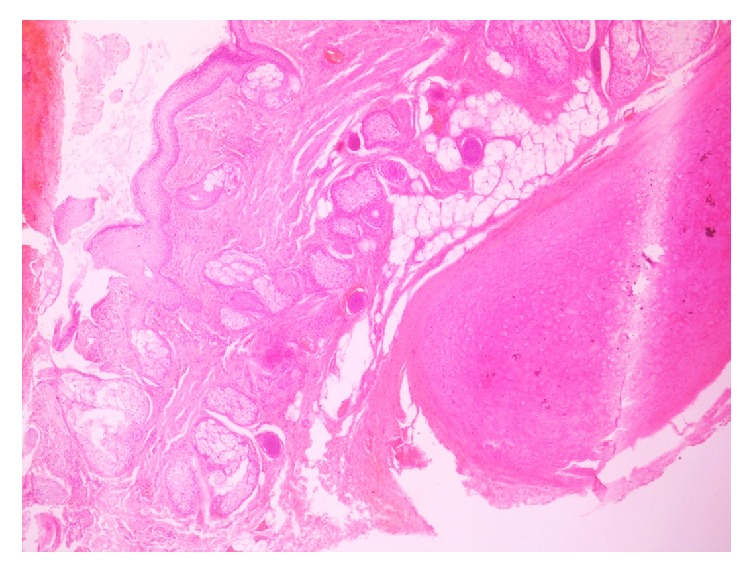
Wall of the cystic cavity is composed of sebaceous glands, adipose tissue, salivary gland tissue, and hyaline cartilage.

**Figure 4:**
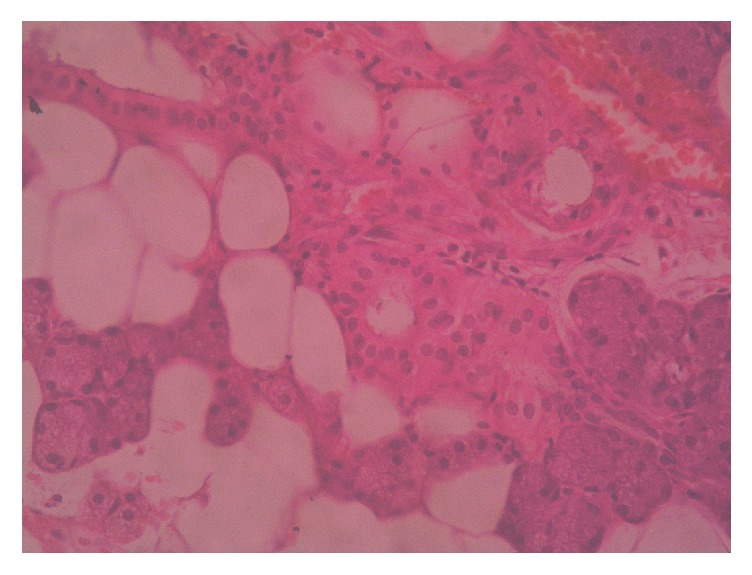
Wall of the cystic cavity is composed of sebaceous glands, adipose tissue, salivary gland tissue, and hyaline cartilage.

With diagnostic and therapeutic aim, complete excisional biopsy of the mass was done as an outpatient procedure under local anesthesia by ENT surgeons. The mass was histopathologically analyzed by pathologists in the department of pathology. Gross examination of the cut-open mass revealed unilocular cystic cavity filled with thick, cheesy material. Hair shafts were not present. Microscopic examination of the mass showed cystic cavity lined by keratinized stratified squamous epithelium (Figures 1 and 2). Underlying wall was composed of sebaceous glands, adipose tissue, salivary gland tissue, and hyaline cartilage (Figures 3 and 4). Hair shafts and follicles were absent. Laminated keratin and anucleate squames were present in the epithelium-cavity interface and in the cystic cavity. Dystrophic calcification was present in one focus. Keratin was present in the tissue of the wall in one focus which incited foreign body giant cell reaction and lymphocytic infiltration.

On the basis of the above-mentioned microscopic features, a diagnosis of teratoid cyst was made.

## 3. Discussion

The term dermoid cyst is used in medical science to include all the six types of keratin-filled cysts: epidermoid, dermoid, teratoid, teratoma, keratinous, and trichilemmal cysts [11]. Out of these six, teratoid cyst has been observed to be very rare.

The search on PubMed with key words “teratoid cyst” yielded 260 results. Out of them, only 76 were related to teratoid cysts. Oral cavity was found to be the most common location [7, 12–15]. A search in the DOAJ (Directory of Open Access Journals) yielded only six results out of which only one was about teratoid cyst in a site other than postauricular region [7].

Other locations where these cysts have been reported in are mandible, submental region, large intestine, mediastinum, thymus, nasopharynx, spinal cord, kidney, and the eyes [16–19].

Another search on PubMed with keywords “teratoid cyst in postauricular region” yielded zero results.

A search in google scholar with key words “post auricular teratoid cyst” yielded one result of teratoid cyst. A 65-year-old female presented with mass in the external auditory meatus (laterality not mentioned). The growth filled the whole meatus [20].

A study of cutaneous cysts in the head and neck region carried out in Jordan analyzed data of 12-year period. The most common location was the scalp which was affected predominantly by pilar cysts. Neck, cheeks, periauricular area, and nasal area were the sites affected by epidermoid cysts in decreasing order of frequency. Dermoid cysts were most commonly found in the periorbital region. Not a single dermoid cyst was found in any sites in the head and neck region including the postauricular region [21].

Another study done in Turkey over a period of five years analyzed 164 cases of cutaneous cysts in the head and neck region. Among cutaneous cysts, epidermoid, dermoid, and trichilemmal cysts were common in descending order of frequency. Teratoid cysts were not observed in the series. There were no cysts of any type in the postauricular region [22].

A systematic search was also made for case reports on teratoid cyst on EMBASE, Cochrane library, MEDION, Retina Medical Search, Scopus, and DARE databases. There were no case reports on teratoid cysts in those databases.

## 4. Conclusion

While there have been several case reports in other sites, this is the first ever reported case of teratoid cyst in the postauricular region. On the basis of review of literature, it is known that other lesions are more common in that location, dermoid cysts other than teratoid cysts and branchial cysts, for example.
